# Usability Comparison Among Healthy Participants of an Anthropomorphic Digital Human and a Text-Based Chatbot as a Responder to Questions on Mental Health: Randomized Controlled Trial

**DOI:** 10.2196/54581

**Published:** 2024-04-29

**Authors:** Almira Osmanovic Thunström, Hanne Krage Carlsen, Lilas Ali, Tomas Larson, Andreas Hellström, Steinn Steingrimsson

**Affiliations:** 1 Region Västra Götaland Psychiatric Department Sahlgrenska University Hospital Gothenburg Sweden; 2 Section of Psychiatry and Neurochemistry Institute of Neuroscience and Physiology, Sahlgrenska Academy University of Gothenburg Gothenburg Sweden; 3 Region Västra Götaland Centre of Registers Gothenburg Sweden; 4 Institute of Health Care Sciences Centre for Person-Centred Care, Sahlgrenska Academy University of Gothenburg Gothenburg Sweden; 5 Centre for Person-Centred Care University of Gothenburg Gothenburg Sweden; 6 Department of Technology Management and Economics Chalmers University of Technology Gothenburg Sweden

**Keywords:** chatbot, chatbots, chat-bot, chat-bots, text-only chatbot, voice-only chatbot, mental health, mental illness, mental disease, mental diseases, mental illnesses, mental health service, mental health services, interface, system usability, usability, digital health, machine learning, ML, artificial intelligence, AI, algorithm, algorithms, NLP, natural language processing

## Abstract

**Background:**

The use of chatbots in mental health support has increased exponentially in recent years, with studies showing that they may be effective in treating mental health problems. More recently, the use of visual avatars called digital humans has been introduced. Digital humans have the capability to use facial expressions as another dimension in human-computer interactions. It is important to study the difference in emotional response and usability preferences between text-based chatbots and digital humans for interacting with mental health services.

**Objective:**

This study aims to explore to what extent a digital human interface and a text-only chatbot interface differed in usability when tested by healthy participants, using BETSY (Behavior, Emotion, Therapy System, and You) which uses 2 distinct interfaces: a digital human with anthropomorphic features and a text-only user interface. We also set out to explore how chatbot-generated conversations on mental health (specific to each interface) affected self-reported feelings and biometrics.

**Methods:**

We explored to what extent a digital human with anthropomorphic features differed from a traditional text-only chatbot regarding perception of usability through the System Usability Scale, emotional reactions through electroencephalography, and feelings of closeness. Healthy participants (n=45) were randomized to 2 groups that used a digital human with anthropomorphic features (n=25) or a text-only chatbot with no such features (n=20). The groups were compared by linear regression analysis and *t* tests.

**Results:**

No differences were observed between the text-only and digital human groups regarding demographic features. The mean System Usability Scale score was 75.34 (SD 10.01; range 57-90) for the text-only chatbot versus 64.80 (SD 14.14; range 40-90) for the digital human interface. Both groups scored their respective chatbot interfaces as average or above average in usability. Women were more likely to report feeling annoyed by BETSY.

**Conclusions:**

The text-only chatbot was perceived as significantly more user-friendly than the digital human, although there were no significant differences in electroencephalography measurements. Male participants exhibited lower levels of annoyance with both interfaces, contrary to previously reported findings.

## Introduction

Conversational user interfaces, also known as chatbots, have been a part of human-computer interactions since the 1960s. Most notable and one of the earliest examples is the ELIZA system, which aimed at simulating a human psychologist [1,2]. A subsequent system named PARRY was implemented in 1972, in which the conversational agent was designed to emulate a patient experiencing schizophrenia [3]. It is by no means a coincidence that the 2 earliest systems targeting the replication of human behavior through natural language processing were both derived from the field of psychiatry. The use of conversational agents has increased exponentially in the past decade [4]. With the availability of systems and the increasing need for 24-hour availability due to globalization, Radziwill and Benton [5] found that perhaps as many as 1 of 3 web-based conversations were conducted with a chatbot or a system moderated by language models, of which some have garnered more than 100 million users [4-8].

Previous research on rule-based conversational agents has shown promise with respect to the alleviation of mental health problems [4,9-11]. In a study by Oh et al [12], patients with panic disorder were randomized to support via a chatbot or support via a self-help book. The patients who were assigned to a chatbot as a support system for exercises in cognitive behavioral therapy were more likely to show symptom alleviation [12]. Digital evaluations as well as digital deliverance of mental health aid were more intensively explored following the COVID-19 pandemic [13]. In a study by Islam et al [14], the authors explored a similar design to that of Oh et al [12] and randomized a set of participants to either book or chatbot intervention for support regarding mental health issues [14]. The group of participants allocated to the chatbot intervention also significantly improved control of helplessness and social phobia scores. Some studies have shown that even a single exposure to a chatbot therapist can have a positive influence on the current state of well-being and repeated exposure can be a good complementary treatment for anxiety [9,10,15,16].

In recent years, a novel facet has been introduced into the evaluative framework for mental health chatbots: the incorporation of voice-controlled visual avatars embodying humanoid countenances colloquially referred to as “digital humans.” These digital entities harness the power of machine learning, emotion-infused linguistics, and adept emulation of facial expressions to cultivate a profound emotional rapport with their users. Research shows that human features elicit more social engagement and can trigger a stronger emotional bond [17]. This has primarily been measured through electroencephalography (EEG) with a specific focus being placed on the importance of increased α and θ wave activity as indicators of overall emotional stability and positive response to stimuli [18-20], while β wave activity has largely been associated with less desirable states of mind such as anxiety and an active stress response [19,21].

While acknowledging the inherent complexity of brain states and wave activity, delving into the extent to which distinct brain wave frequencies exert influence during a chat session presents an intriguing avenue for investigating the emotional states of the user. In a study by Bos et al [22], the authors explored capturing vigilance and states of emotion with EEG in usability testing of chatbot technology. The study findings revealed that EEG effectively captured the facets of user experience and conversation that piqued interest. This was accomplished through the delineation of γ wave activity, predominantly linked with positivity and problem-solving. Consequently, this approach affords researchers a more objective means of apprehending user experience. A study by Ciechanowski et al [23] indicated that there is a difference in emotional response and usability preference between text-based chatbots and digital humans, with text-based chatbots eliciting more positive interactions.

Although EEG has served as a proficient tool for quantifying objective assessments of emotional responses to chatbot interventions, it is customary to use usability scales for capturing the subjective dimensions pertaining to emotions and experiences in the context of chatbot systems. While a universally accepted benchmark for conducting usability tests on chatbots remains elusive, numerous studies have gravitated toward the adoption of the System Usability Scale (SUS-10) [24-29] and the Speech User Interface Service Quality scale. SUS-10 captures the overall usability of a system independently of the platform or interface. The score ranges from 0 to 100, indicating higher usability with increasing scores [26]. A score of 68 is considered as a passing grade, while a score below 50 is considered as indicating that the system has less optimal usability. For a system to be considered as exceptionally good in terms of its design and usability, a score of 85 on average should be applied [29-31]. In the previously mentioned study by Oh et al [12], mean SUS-10 was not significantly worse or better comparing a chatbot and a book: 64.5 (SD 17.0) versus 69.5 (SD 17.2), respectively (*P*=.35). Several studies have advocated the idea that chatbots represent user-friendly alternatives to conventional analog methods or standard digital tools, such as forms [4]. Nonetheless, there is research that suggests the design flaws in a chatbot system can markedly diminish its effectiveness, potentially leading to perceptions of unhelpfulness among users [3]. Chatbots that are perceived as unhelpful, repetitive, or lacking the users’ trust tend to receive a lower SUS-10 score [32].

Many social chatbots aim to comfort, support, and advise their users [3]. Studies show that the availability of chatbot technology is what is central to its perception of usefulness compared with human therapists. However, studies have also noted that most users prefer human therapists and are more interested in using the system as a complementary tool when a human therapist is not available [33-35]. While mental health chatbots are generally viewed positively by the user, there are many issues that can lead to decreased usability, lower SUS-10 scores, and undesirable outcomes such as irritation or worsened mental health. The propensity for misunderstanding, miscommunication, and annoyance are frequently reported in qualitative assessments of social support chatbots [33-35]. Feeling annoyed by repetitive messaging, incoherent conversations, and inability to comprehend the user’s needs are frequently named as issues that increase the feeling of annoyance in users of social support chatbots [34]. The selection of an interface can wield a considerable influence on both the effectiveness and user-friendliness of a system. Users exhibit disparate reactions to chatbots depending on whether they incorporate an avatar, particularly one with humanoid attributes capable of evoking emotions. Although our understanding of chatbot usability and user preference is somewhat limited, investigations into anthropomorphic interfaces do underscore their ability to affect our emotional states [36].

The chatbot used in our study, known as BETSY (Behavior, Emotion, Therapy System, and You), uses 2 distinct interfaces: a digital human, voice-activated user interface with anthropomorphic features and a text-only user interface. Within the scope of our investigation, we aimed to thoroughly examine both interface modalities. Phase 1 of usability testing involves enlisting the participation of healthy volunteers.

The aim of this study was to explore to what extent a digital human and a text-only chatbot interface differed in usability when tested by healthy participants. We also set out to explore how chatbot-generated conversations on mental health (specific to each interface) affected self-reported feelings and biometrics.

## Methods

### Construction of the User Interface (BETSY)

This project adopted a participatory design approach to ensure the broad involvement of health care professionals, patients, and the public. A multidisciplinary team consisting of 2 psychiatrists, 2 psychiatric nurses, 4 clinical psychologists, 1 user of health care services, and 1 engineer was assembled to comprehensively address ethical, medical, and legal considerations for a potential chatbot. Team members were selected for their expertise in digitalization and psychiatry. Before the initial workshop, where the algorithm’s preliminary outline was presented, the engineer created a survey. This survey drew partly from Radziwill and Benton [5] quality attribute listing, which synthesized findings from various chatbot usability projects.

A survey was distributed to the public via the secure research platform Psytoolkit.org, offering heightened anonymity by omitting the collection of metadata such as IP addresses and locations. The survey comprised 8 multiple-choice questions and 4 open-ended free-text questions, covering demographic information, design requirements, functionality suggestions, and overall attitudes toward mental health chatbots. It was accessible for 14 days and disseminated through various social media channels. Subsequently, the collected data were analyzed to inform a series of 4 workshops conducted by the group between June 2020 and December 2020. During these workshops, the chatbot’s design, encompassing appearance, content, and personality, underwent iterative development based on input from the general public and co-designer feedback, with the latter representing a patient perspective. A comprehensive account of this process will be available in a separate publication.

**Figure 1:**
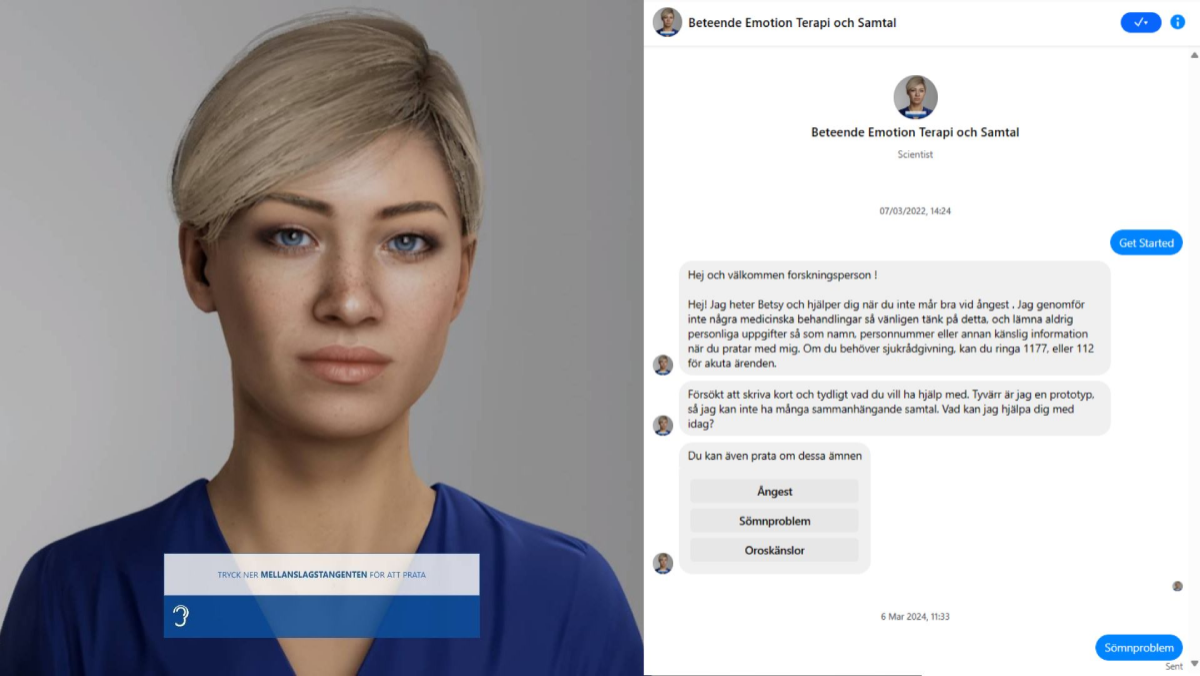
Two versions of BETSY (Behavior, Emotion, Therapy System, and You): digital human, voice-activated (left) and text-only (right).

Two versions of the chatbot (Figure 1) were created: one enabling voice interaction with a facial expression and an avatar component, and another relying solely on text-based communication with an avatar image. The digital human was implemented using Dialogflow (Google) for conversation logic and connected to the UNEEQ platform for the human-avatar interface. Data infrastructure was hosted by Deloitte Digital and VästraGötalandsregionen/VGR-IT. In contrast, the text-only BETSY chatbot was developed on the Itsalive.io platform and deployed to a research and development account on Facebook that was closed to the public. Importantly, no personal metadata were collected during on-site testing via digital platforms. The users did not use their personal social media accounts to talk to the chatbot.

Both versions of BETSY encompassed 24 topics (detailed in Multimedia Appendix 1) related to mental health, including anxiety, depression, stress, sleep, addiction, eating disorders, anger, hopelessness, helplessness, loneliness, sadness, suicidal ideation, and suicidality, among others. These chatbots were designed in the Swedish language. An assessment was conducted to evaluate the alignment of the text-only and digital human algorithms. Specifically, testers posed identical questions to both systems within various domains, with only 1 instance revealing a discrepancy when the digital human could not provide an appropriate response while the text-based bot could, indicating the need for further refinement.

### Recruitment

In this initial phase of system exploration, our focus was on evaluating the system’s capabilities using volunteers who did not exhibit severe anxiety. As the system is still in its prototype stage, we exercised caution to avoid any potential exacerbation of symptoms in individuals with severe anxiety. Our recruitment announcement, disseminated through various social media channels associated with Sahlgrenska University’s official account, specified that participants should be 18 years or older, free from any current mental health disorders, and willing to physically attend the testing facility in Gothenburg, Sweden.

### Participants

**Figure 2:**
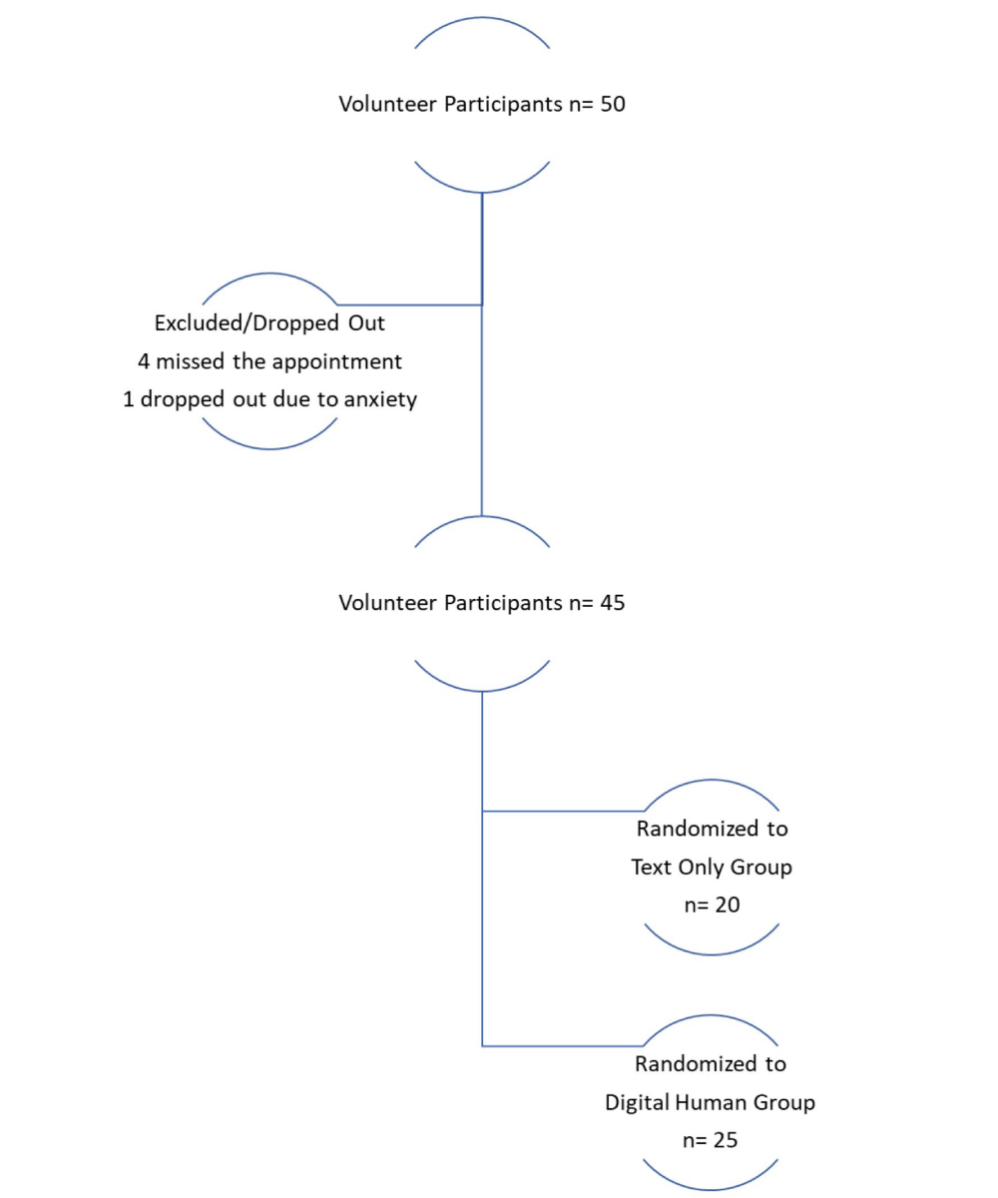
Flow chart of participation and exclusion.

Of the 50 individuals who initially volunteered, 5 participants (2 men and 3 women) opted out before providing their consent (Figure 2). Subsequently, 45 individuals attended the screening at the test facility. Each participant was required to provide informed consent before undergoing the Generalized Anxiety Disorder (GAD-7) scale assessment for anxiety symptoms. Those scoring 14 or higher on the GAD-7 were excluded from the study (Figure 2). Eligible participants were then randomly assigned to one of two groups: (1) engaging in text-based conversations with the text-only BETSY or (2) participating in voice-based interactions with the digital human (Figure 2).

The randomization process was conducted with strict double-blind procedures overseen by an independent researcher not affiliated with this project and facilitated by an automated randomization system, ensuring the impartiality of the allocation.

### Prechat Procedure

The experiments were performed during the COVID-19 pandemic (June 2021-November 2021). Due to safety precautions, the participants were greeted by a tester wearing protective gear, including a surgical R-II mask, gloves, a face visor, and hospital scrubs. Protective gear was also offered to participants upon their arrival. Participants were then placed in a sanitized room equipped with a screen, which underwent thorough sanitization with medical-grade disinfectants and a sterilizing UV lamp before and after each participant’s session.

Before starting the chat with BETSY, participants were outfitted with a mobile dry-sensor EEG device to record their brain wave activity. Additionally, their blood pressure and pulse were recorded on the left arm after a 5-minute seated rest. Systolic and diastolic blood pressure were measured using a digital sphygmomanometer, and the pulse was monitored with a pulse oximeter.

Despite relatively relaxed COVID-19 restrictions during the testing period, the tester opted not to be physically present in the room to minimize any potential risk of contagion. Each participant sat alone in the room, with the tester observing remotely via a nonrecordable streaming camera. This camera served to facilitate real-time communication and allowed the tester to monitor the participant’s reactions and anticipate any need for assistance. The participants were made aware of this procedure.

Following the measurement of biometric data (blood pressure and pulse), participants were instructed to complete a questionnaire. This questionnaire covered their prior experiences with mental health chatbots as well as their demographic information, including sex, occupation, and marital status. Additionally, participants rated their overall well-being on a visual analog scale ranging from 1 (not good at all) to 10 (feeling excellent) before starting their session with BETSY. Participants were given instructions by the tester along with an accompanying sheet that provided potential chat scenarios and specified the topics within BETSY’s scope. Each participant had a maximum of 30 minutes to engage with the chatbot version they were assigned to.

### Chat Session Protocol and EEG Data Collection Process

EEG recording commenced simultaneously with the participant’s initiation of their session with BETSY. We used a dry-sensor mobile EEG system, specifically the MUSE headband from Interaxon, which incorporates 7 sensors. These sensors include 3 frontal reference sensors and active sensors situated at Fp1, Fp2, Tp9, and Tp10.

The MUSE headband was seamlessly connected to a smartphone via Bluetooth and data collection was facilitated through the Mind Monitor app on an Android smartphone. It is noteworthy that this app neither necessitates user registration nor collects data that can identify or pinpoint individual users or their locations. Consequently, the data were recorded in an anonymous fashion and stored as a CSV file within the smartphone’s document section.

Upon placing the EEG headband on the participant, they were requested to close their eyes to enable the Mind Monitor to perform calibration. After calibration was successfully established, the participant was left alone in the room to initiate a conversation with BETSY. Importantly, the EEG recording remained active throughout the entire conversation and was terminated when the participant indicated they had concluded their interaction with the chatbot.

### Postchat Procedure

Upon reaching a point of satisfaction with the conversation or upon the completion of their allocated chat time, participants were directed to complete supplementary questionnaires and scales. Participants were administered the SUS-10, developed by Lewis and Sauro [26]. The SUS-10 calculates an average score derived from a 10-item questionnaire with response options ranging from 0 to 4, resulting in a total score between 0 and 100, as outlined by Bangor et al [31].

Participants were also presented with multiple-choice questions regarding their emotional state during the chat. Additionally, in the digital human group, participants were instructed to fill out the Standardized Questionnaires for Voice Interaction Design Short Version (SUISQ-MR). SUSIQ is a questionnaire tailored to assessing critical usability attributes of Interactive Voice Response, as outlined by Lewis and Sauro [26]. The original scale comprises 25 items categorized into 4 factors: user goal orientation, customer service behaviors, speech characteristics, and verbosity. SUISQ-MR, a shortened version, encompasses 9 items rated on a 7-point Likert scale, ranging from “strongly disagree” to “strongly agree.” Higher scores on this scale indicate a more favorable assessment of the system’s usability [24].

Furthermore, participants were provided with an open-ended questionnaire to gather their suggestions and insights regarding their session experience. It should be noted that qualitative data from this survey will be reported separately.

### EEG Monitoring and Analysis

The monitoring of EEG activity entails the use of the Mind Monitor app, which captures and visually represents EEG brain wave data. The quantification of absolute brain wave values is predicated on the computation of absolute band powers. These powers are derived from the logarithm of the power spectral density calculated from the EEG data for each channel.

The frequency spectrum categories used for this analysis encompass the following bandwidths: δ (1-4 Hz), θ (4-8 Hz), α (7.5-13 Hz), β (13-30 Hz), and γ (30-44 Hz). Notably, the EEG power spectral density values acquired from the sensors typically fall within the {–1:+1} range, which is subsequently transformed into a more intelligible {0:100} range for text-based display purposes.

Subsequently, the collected EEG data underwent an analytical process facilitated by the Mind Monitor online graphing tool. Within this tool, the values are presented as average (dB) per session. It is imperative to note that, in the context of this study, we exclusively used absolute data for our analyses (information sourced from Mindmonitor.com and Choosemuse.com).

### Statistical Analysis

All data were entered and processed in SPSS Statistics (version 28.0.1.1; IBM Corp). For group differences, means analysis was used using Pearson *χ*^2^ asymptotic significance (2-sided) set at .05 as the significance level. For continuous outcome variables such as SUS-10, SUISQ-MR, brain wave activity, positivity, and GAD-7, linear regression analyses were used. The data were tested for kurtosis and skewness. Based on the results, *t* tests were performed. All results were analyzed according to group.

### Ethical Considerations

All ethical decisions were guided by the Declaration of Helsinki and its subsequent amendments. The study protocol was reviewed and approved by the central ethical review board; Etikprövningsmyndigheten, Sweden (DRN 2021-02771). All precautions were taken in order to avoid any possible contagion from COVID-19. Due to the nature of the prototype, no patients were used in this initial examination of the chatbot in order to ensure that vulnerable participants would not be negatively affected by errors and flaws that might be present in a prototype-stage system.

## Results

### Characteristics of Participants

There were no statistically significant differences in the demographic variables between the digital human and text-only groups (Table 1). No participants were excluded due to a high GAD-7 score. The age of the participants ranged from 24 to 68 years, and as only 12 participants registered their age, this variable was consequently excluded from more advanced analyses (Table 1).

**Table 1:** Demographic characteristics of the study population.

Characteristic			Text-only, n (%)		Digital human, n (%)		*P* value^a^
**Sex**							.62
	Male	8 (40)		10 (40)			
	Female	12 (60)		15 (60)			
**Marital status**							.49
	Married	11 (55)		11 (44)			
	Single	5 (25)		3 (12)			
	Divorced	1 (5)		2 (8)			
	Domestic partnership	2 (10)		7 (28)			
	Other	1 (5)		2 (8)			
**Educational level**							.74
	High school/trade school	1 (5)		3 (12)			
	Bachelor’s	7 (35)		8 (32)			
	Master’s	7 (35)		11 (44)			
	PhD	3 (15)		1 (4)			
	Other/higher than master’s or PhD	2 (10)		3 (8)			
**Occupation**							.30
	Sick leave/sick leave part-time	0 (0)		1 (4)			
	Working part-time	0 (0)		2 (8)			
	Working full time	18 (90)		19 (76)			
	Student	2 (10)		1 (4)			
	Retired	0 (0)		2 (8)			
**Housing**							.35
	Living alone	4 (20)		2 (8)			
	Cohabitation	16 (80)		23 (92)			

^a^Pearson *χ*^2^ test.

### Comparison Between Digital Human and Text-Only Chatbots

When comparing self-reported emotional states between the digital human and the text-only chatbot groups, it was observed that participants using the digital human exhibited a notably higher propensity to report feelings of nervousness versus the text-only chatbot group (Table 2). The mean GAD-7 score for the text-only chatbot group was 2.32 (SD 2.52) compared with 2.80 (SD 2.60) for the digital human chatbot group, with no statistically significant difference between the groups.

**Table 2:** Self-reported prior therapy experience, emotions, biometrics, and electroencephalography.

		Text-only	Digital human	*P* value^a^	
**Therapy, n (%)**					.47
	Yes	2 (10)	4 (16)		
	No	18 (90)	20 (80)		
	Do not remember	0	1 (4)		
“**Have you talked to a chatbot about mental health before?”, n (%)**					.84
	Yes	1 (5)	1 (4)		
	No	19 (95)	24 (96)		
	Do not remember	0	0		
GAD-7^b^ score, mean (SD)^a^		2.3 (2.5)	2.8 (2.6)	.56	
Positivity toward chatbot, mean (SD)^c^		7.1 (2.1)	7.5 (2.1)	.69	
“**Do you feel closeness to BETSY^d^?”, n (%)**					.46
	Yes	7 (35)	11 (45.8)		
	No	13 (65)	13 (54.2)		
“**Did you feel relaxed?”, n (%)**					.23
	Yes or sometimes	17 (89.5)	17 (73.9)		
	No	2 (10.5)	6 (26.1)		
“**Did you feel nervous?”, n (%)**					.02
	Yes or sometimes	0	6 (26.1)		
	No	19 (100)	17 (73.9)		
“**Did you feel sad?”, n (%)**					.1
	Yes or sometimes	0	3 (13.0)		
	No	19 (100)	20 (87.0)		
“**Did you feel annoyance?”, n (%)**					.8
	Yes or sometimes	9 (47.4)	10 (43.5)		
	No	10 (52.6)	13 (56.5)		
VAS-W^e^ presession, mean (SD)^f^		8.8 (1.32)	8.4 (1.41)	.33	
VAS-W postsession, mean (SD)^f^		8.8 (1.23)	8.3 (1.27)	.14	
Pulse presession, mean (SD)		72.2 (10.7)	71.6 (10.9)	.77	
Pulse postsession, mean (SD)		68.5 (8.8)	70.2 (11.1)	.58	
Average δ wave activity, mean (SD)		114 (30)	97 (25)	.06	
Average θ wave activity, mean (SD)		86 (23)	74 (21)	.08	
Average α wave activity, mean (SD)		97 (27)	82 (24)	.03	
Average β wave activity, mean (SD)		81 (17)	76 (20)	.34	
Average γ wave activity, mean (SD)		65 (15)	66 (21)	.98	
SUS-10^g^, mean (SD)		74.82 (10)	64.80 (14)	.01	
SUISQ-MR^h^, mean (range)		N/A^i^	4.92 (2.83-6.75)	N/A	

^a^Pearson *χ*^2^ test for categorical variables and ANOVA for continuous variables.

^b^GAD-7: Generalized Anxiety Disorder Scale.

^c^Participants were asked to what extent they felt positive about talking to BETSY about mental health with scores ranging from 1 (not positive at all) to 10 (very positive).

^d^BETSY: Behavior, Emotion, Therapy System, and You.

^e^VAS-W: Visual Analogue Scale for Well-Being.

^f^Range from 1 (not feeling well at all) to 10 (feeling very good).

^g^SUS-10: System Usability Scale.

^h^SUISQ-MR: Standardized Questionnaires for Voice Interaction Design Short Version.

^i^N/A: not applicable.

Conversely, the evaluation of system usability as gauged by SUS-10 showed a significant (*P*=.01) difference between the groups. Notably, the mean SUS-10 score was higher in the text-only chatbot group at 75.34 (SD 10.01; range 57-90) compared with the digital human group at 64.80 (SD 14.14; range 40-90). In addition, the digital human group underwent assessment using SUISQ-MR: BETSY had a mean score of 4.92 (SD 0.83; range 2.83-6.75), as depicted in Table 2, which is indicative of a commendable level of usability for BETSY’s voice interface in accordance with the framework presented by Lewis [24].

### Biometric Measures

There were no statistically significant distinctions for mean values of blood pressure or pulse between the groups either at baseline or following exposure to the interventions. Specifically, the mean pulse rate showed no discernible variations between the groups both before and after exposure, reflecting consistent values across the groups on average (data not shown).

The EEG signals collected during the study exhibited suboptimal quality, which was primarily attributed to participant movement and signal acquisition sensitivity. These challenges occasionally disrupted signal continuity during the sessions. Nonetheless, the data yielded adequate information to calculate mean values pertaining to δ, θ, α, β, and γ frequency bands, as facilitated by the web-based graphing module within the MindMonitor’s platform. Only 1 significant difference was found in terms of means: the average α was significantly higher in the text-only group (Table 2).

### System Usability Scale and Outcomes

Predictors of SUS-10 usability were used as a dependent variable in linear regression analysis and matched against biometric and subjective variables. Each variable was independently analyzed in a model together with SUS-10 as the dependent variable. Analysis showed that there was a significant positive relationship between average α and θ wave activity and SUS-10 in the chat-only group. A significant positive relationship was seen between SUISQ-MR scores and SUS-10 (Table 3).

**Table 3:** Linear regression analysis between usability and biometric variables.

		Unstandardized coefficients			*P* value^a^
		β	SE		
**SUS-10^b^ × positivity**					
	Text (n=19)	1.82	1.02	.09	
	Voice (n=24)	1.313	1.361	.35	
**SUS-10 × average** **δ** **wave activity**					
	Text (n=17)	0.153	0.08	.07	
	Voice (n=23)	0.062	0.12	.61	
**SUS-10 × average** **θ** **wave activity**					
	Text (n=17)	0.212	0.1	.05	
	Voice (n=23)	0.083	0.146	.57	
**SUS-10 × average** **α** **wave activity**					
	Text (n=17)	0.196	0.083	.03	
	Voice (n=23)	0.054	0.124	.67	
**SUS-10 × average** **β** **wave activity**					
	Text (n=17)	0.251	0.143	.10	
	Voice (n=23)	0.03	0.152	.85	
**SUS-10 × average** **γ** **wave activity**					
	Text (n=17)	0.148	0.177	.42	
	Voice (n=23)	–0.017	0.146	.91	
SUS-10 × SUISQ-MR^c^ (n=24)		8.100	2.976	.01	

^a^Pearson *χ*^2^ test.

^b^SUS-10: System Usability Scale.

^c^SUISQ-MR: Standardized Questionnaires for Voice Interaction Design Short Version.

### Self-Reported Feelings and Gender

Furthermore, our investigation sought to discern whether significant gender disparities existed in terms of self-reported emotions. Notably, we observed a significant difference between men and women, with men exhibiting a notably lower tendency to report feeling annoyed by BETSY in contrast to women. No other statistically significant distinctions were identified (Table 4).

**Table 4:** Sex difference in emotional expression toward BETSY (Behavior, Emotion, Therapy System, and You).

Self-reported feeling				Men, n (%)		Women, n (%)		*P* value^a^
**Did you feel annoyed?**								
	**Chat**							.03
		Yes	1 (11)		8 (67)			
		No	6 (88)		4 (33)			
	**Voice**							.03
		Yes	1 (12.5)		9 (60)			
		No	7 (84.5)		6 (40)			
**Did you feel relaxed?**								
	**Chat**							.68
		Yes	6 (86)		11 (92)			
		No	1 (14)		1 (8)			
	**Voice**							.29
		Yes	7 (87.5)		10 (67)			
		No	1 (12.5)		5 (33)			
**Did you feel closeness or connection to BETSY^b^?**								
	**Chat**							.25
		Yes	4 (50)		3 (25)			
		No	4 (50)		9 (75)			
	**Voice**							.63
		Yes	4 (40)		7 (50)			
		No	6 (60)		7 (50)			
**Did you feel nervous?**								
	**Chat**							
		Yes	N/A^c^		N/A			
		No	7 (100)		12 (100)			
	**Voice**							.93
		Yes	2 (25)		4 (27)			
		No	6 (75)		11 (73)			
**Did you feel sadness?**								
	**Chat**							
		Yes	N/A		N/A			
		No	7 (100)		12 (100)			
	**Voice**							.17
		Yes	0		3 (20)			
		No	8 (100)		12 (80)			

^a^Pearson *χ*^2^ test.

^b^BETSY: Behavior, Emotion, Therapy System, and You.

^c^N/A: not applicable.

An analysis of feelings of closeness and positivity toward chatbot conversations was undertaken to explore differences between men and women. In mean score analyses, the results showed that men were significantly more positive toward talking to BETSY prior to the session: 8.16 (SD 1.50) for men and 6.81 (SD 2.30) for women (*P*=.34). Conversely, there were no discernible gender-based differences concerning feelings of closeness during chatbot interactions.

## Discussion

### Principal Findings

This study explored how a digital human versus text-only chatbot interface affected usability and user experience in healthy participants. We also examined how chatbot-generated conversations on mental health affected self-reported feelings and biometrics. The overall sample was small and, thus, should not act as a point of reference for generalization. This study was, however, not smaller than the average study in the investigative field of mental health chatbots [9,10,12,37-41].

While the text-only system scored higher on usability, both versions of the chatbot scored average or above average with respect to overall usability [31]. The mean text-only chatbot SUS-10 score of 75.34 falls between the threshold of good (a score of 70) and excellent (a score of 80 and above) [29-31]. However, the score for the digital human (64.8) indicates that the system is perceived to be usable, but has room for improvement. Usability can be affected by many factors such as user interface design, content layout, and overall user experience [42,43].

The digital human score indicates that there may be areas for improvement in terms of all of the aforementioned aspects. It should also be noted that the SUS-10 scale does not measure a specific feature or aspect of system design, but instead provides an overall assessment of user experience [31]. Using more elaborate scales that cover more dimensions across the system is more suitable for a more in-depth analysis of the usability of chatbots. It can also be noted that the range of scores was much higher for the text-only interface (lowest score for the text-only group was 57 and the equivalent for the voice-only chatbot was 40), which indicates much poorer usability.

Taking into account the specific usability of the digital human interface, usability was considered high with an average SUISQ-MR score of 4.92. This score indicates that the voice interaction design is likely to be perceived as intuitive and useful by users. A score of 4.92 falls within the range of 4.5-5.5, which has been classified as “very good” in previous studies [24]. In addition, higher scores on the SUISQ-MR have been associated with increased user engagement and task completion rates. Therefore, a score of 4.92 can be interpreted as an indication that the voice interaction design will likely provide positive user experiences [24].

Men were more likely to score higher on positivity and less likely to report feeling annoyed by BETSY independently of the interface, which is the opposite of other studies that indicate men are more likely to be annoyed or aggressive toward female avatars [44]. In a study by Luger and Sellen [45], the authors found that higher expectations of the system lead to a higher risk of disappointment and lower scores: this could possibly explain why female participants were more agitated as their expectations might have been higher [45], however, we have no data to explore this empirically in the frame of this study. Unlike other studies with similar designs and populations [40], we did not analyze the content of the conversations. The conversations between BETSY and the participants were deleted immediately after the session as the research question was geared toward usability and not the effect on the user’s own mental health status. Much like the results from Hearst and Tory [46], the interface was the focus of this investigation. Hearst and Tory showed that a well-designed conversation tamped the choice of interface. The interface played into the perception of usability only when the system failed to respond or create barriers to conversation. In our study, we used biometric data to explore feelings of relaxation or excitement/agitation while using BETSY. Despite EEG data collection not being optimal, we were able to collect and compare some brain wave activity data in the study groups during the sessions. Even though the amplitude of brain wave activity can result in large intraindividual variation, the data were evenly distributed and there were no mean differences between the groups in our study. While we observed no significant association between scores of usability and β wave activity (more likely to be associated with frustration, agitation, or perhaps excitement), we did observe brain wave activity that is typically associated with relaxed states of mind [18-21] and this had a positive linear relationship with SUS-10 score. This indicated that the chat-only group was either more relaxed or less aroused (or both). The explanation could lie in the combination of the small sample and the fact that more individuals in the voice-only chat group reported feeling nervousness, a feeling that generally elicits higher brain wave activity and less relaxation [20,47]. With the low quality of data, combined with a limited sample, it is hard to draw any generalizable conclusions from the biometric data.

Feelings of closeness did not differ between the 2 interfaces and seem not to have been affected by the presence of anthropomorphic features. When gender was explored as a factor, there was no significant difference to what extent men and women reported feeling close to BETSY in the respective assigned interfaces. Due to the small sample size in our study, it was not possible to perform further and more elaborate designs looking at mediation of other demographic or biometric factors in a reliable way.

When devising chatbots for mental health, this study indicates that a mixed approach might be the best course of action, allowing the user to choose a preferred way of interacting with the chatbot.

### Limitations

This study consisted of healthy volunteers. It is good to keep in mind that mental health issues can affect some parts of cognitive performance [48] and, thus, usability may not be equally perceived by a person in a state of emotional distress and a healthy volunteer. Further investigation and collaboration are needed in future studies to capture the usability aspects of individuals who are in an active state of distress.

The results of this study suggest that overall usability seems to be perceived as higher for the text-only chatbot interface and no significant emotional boost was present with the addition of anthropomorphic features to a digital human chatbot.

Further studies which include a larger sample of participants as well as participants who experience mild to moderate anxiety are needed to explore and further evaluate the research question posed in this paper. In this study, the age range was limited, and the variable was incomplete. In future studies, we will strive to include more young adults and adults older than 60 years.

Large language models and application programming interface models were not available at the time this chatbot was constructed and neither were Metahuman creator or more advanced voice-cloning or voice-generating options, which would have significantly improved the anthropomorphic features of the digital human. The first iteration of the generative pretrained transformer was not available to the public and the generative pretrained transformer-3 application programming interface had a limited release during the development of this project: it was not available to our team until a year after the project was completed. With large language models, repetitiveness and limitations in terms of variability of answers would have most likely been avoided; however, the aim of our investigation was not the general effect of the content but rather the perception of text- versus voice-driven interfaces.

### Conclusions

In conclusion, the text-only chatbot was perceived as more user-friendly in terms of usability indicators for SUS-10. However, both the digital human and text-only interfaces scored average or above average in comparison to other studies performed on mental health chatbots. Although biometric data did not differ significantly, we saw significant gender differences in terms of prechat positivity and postchat annoyance, which is contrary to other studies. Male participants in our study were more likely to report higher prechat positivity toward BETSY and report less irritation postchat. SUISQ-MR also indicated that BETSY’s overall usability and voice were highly ranked compared with other studies, indicating that there is great promise for mental health chatbots independently of the chosen user interface.

## Appendix

Multimedia Appendix 1 CONSORT-EHEALTH checklist (V 1.6.1).

## Abbreviations

BETSY: Behavior, Emotion, Therapy System, and You
EEG: electroencephalography
GAD-7: Generalized Anxiety Disorder-7
SUS-10: System Usability Scale-10
SUISQ-MR: Standardized Questionnaires for Voice Interaction Design Short Version
