# Supplementary material for: Usability Comparison Among Healthy Participants of an Anthropomorphic Digital Human and a Text-Based Chatbot as a Responder to Questions on Mental Health: Randomized Controlled Trial
Source: JMIR Hum Factors. 2024 Apr 29;11:e54581. doi: 10.2196/54581 (PMC11091805; doi:10.2196/54581)
Supplement: Multimedia Appendix 1 [file humanfactors_v11i1e54581_app1.pdf]

CONSORT-EHEALTH Checklist V1.6.2 Report	Manuscript Number	54581
**(based on CONSORT-EHEALTH V1.6), available at [http://tinyurl.com/consort-ehealth-v1-6].**		
**Date completed**		
6/7/2049 0:00:00		
**by**		
Almira Osmanovic Thunström		
Feasibility of a Voice-only Chatbot with Anthropomorphic Features Compared to a Text-based Chatbot as a Responder to Questions on Mental Health: a Randomized, Controlled Trial among Healthy Participants		
TITLE		
**1a-i) Identify the mode of delivery in the title**		
The title provides a clear and concise description of the study without using any ambiguous terms. It also includes all of the required information about the condition, target group, and mode of delivery. Therefore, the title complies with the CONSORT checklist item regarding mode of delivery.		
**1a-ii) Non-web-based components or important co-interventions in title**		
The title does not mention any non-web-based components.		
**1a-iii) Primary condition or target group in the title**		
The title clearly identifies the condition (mental health) and the target group (healthy participants).		
ABSTRACT		
**1b-i) Key features/functionalities/components of the intervention and comparator in the METHODS section of the ABSTRACT**		
"We explored to what extent a voice-only chatbot with anthropomorphic features differed from a traditional text-only chatbot regarding perception of usability though the System Usability Scale (SUS-10), emotional reactions though electroencephalography, and feeling of closeness. Healthy participants (n=45) were randomized to two groups that used a voice-only chatbot with anthropomorphic features (n=25) or a text-only chatbot with no such features (n=20). The groups were compared by linear regression analysis and t-tests."		
**1b-ii) Level of human involvement in the METHODS section of the ABSTRACT**		
The abstract does not explicitly state whether the chatbots were fully automated or therapist-assisted. However, it is highly unlikely that a chatbot is human operated. In such case, a human take-over protocol is to be described and is a deviation from standard use of the term chatbot.		
**1b-iii) Open vs. closed, web-based (self-assessment) vs. face-to-face assessments in the METHODS section of the ABSTRACT**		
The abstract states that the outcomes were self-assessed through questionnaires, it also states that the target was healthy volunteers, however, the abstract does not specify that which platform i.e. online was used to recruit participants. Thus will be added in a revision to comply with the checklist.<br><br>"....We also set out to explore how chatbot-generated conversations on mental health (specific to each interface) affected self-reported feelings and biometrics."<br><br>"We explored to what extent a voice-only chatbot with anthropomorphic features differed from a traditional text-only chatbot regarding perception of usability though the System Usability Scale (SUS-10)"		
**1b-iv) RESULTS section in abstract must contain use data**		
The abstract complies with checklist item.<br><br>The abstract states that 45 healthy participants were randomized to either the voice-only chatbot group (n=25) or the text-only chatbot group (n=20).<br><br>The abstract also states that the SUS-10 scores were not significantly different between the two groups (mean SUS-10 score for text-only chatbot = 75.34, mean SUS-10 score for voice-only chatbot = 64.80).<br><br>The abstract also states that women were more likely to report feeling annoyed by the voice-only chatbot.		
**1b-v) CONCLUSIONS/DISCUSSION in abstract for negative trials**		

The subitem is present in the abstract. There is a clear demonstration of outcome.<br>"No differences were observed between the text-only and voice-only group regarding demographic features. Mean (SD) SUS-10 score was 75.34 (10.01) [range, 57-90] for text-only chatbot versus 64.80 (14.14) (range, 40-90) for the voice-only chatbot. Both groups scored their respective chatbot interfaces as average or above average in usability. Women were more likely to report feeling annoyed by BETSY."		
INTRODUCTION		
**2a-i) Problem and the type of system/solution**		
The paper follows the subitem.<br><br>Problem and Background: The demand for mental health services is increasing, but access to traditional therapy is often limited.<br><br>e.g. "Due to the nature of the topic, usefulness is more central to chatbots in mental health care than, for example, customer service. Many social chatbots aim to comfort, support, and advise their users [3]. Studies show that the availability of chatbot technology is what is central to its perception of usefulness compared to human therapists. However, studies have also noted that most users prefer human therapists and are more interested in using the system as a complementary tool when a human therapist is not available [33-35]"<br><br>The introduction describes the problem of mental health and the increasing use of chatbots as a way to address this problem. The introduction mentions that the study is comparing two types of chatbot interfaces: a voice-only interface with anthropomorphic features and a text-only interface.<br><br>The introduction mentions that the goals of the study are to explore the difference between the two chatbot interfaces in terms of usability and effect on self-reported feelings and biometrics.<br><br>The introduction does not however explicitly state whether the chatbot is intended to be a stand-alone intervention or incorporated into a broader health care program nor does it explicitly state whether the chatbot is intended for a particular patient population. This should be added in a revision.		
**2a-ii) Scientific background, rationale: What is known about the (type of) system**		
Background and rational are covered through noting that:<br><br>e.g. "While mental health chatbots are generally viewed positively by<br>the user, there are many issues that can lead to decreased usability, lower SUS-10 scores, and undesirable outcomes such as irritation or worsened mental health. Propensity for misunderstanding, miscommunication, and annoyance are frequently reported in qualitative assessments of social support chatbots [33-35]. Feeling annoyed by repetitive messaging, non-coherent conversations, and inability to comprehend the user's needs are frequently named as issues which increase the feeling of annoyance in users of social support chatbots [34]. The selection of an interface can wield a considerable influence on both the effectiveness and user-friendliness of a system. Users exhibit disparate reactions to chatbots depending on whether they incorporate an avatar, particularly one with humanoid attributes capable of evoking emotions."		
**Does your paper address CONSORT subitem 2b?**		
The study has a specific objective and thus follows this subitem. The introduction states that the objective of the study is to explore the difference between a voice-only and a text-only chatbot interface in terms of usability and effect on self-reported feelings and biometrics.<br><br>"The aim of this study was to explore to what extent a voice-only and a text-only chatbot interface differed on usability when tested by healthy participants. We also set out to explore how chatbotgenerated conversations on mental health (specific to each interface) affected self-reported feelings and biometrics"		
METHODS		
**3a) CONSORT: Description of trial design (such as parallel, factorial) including allocation ratio**		

The trial design is stated in the methods section.<br><br>The study employed a parallel-group randomized controlled trial design, comparing two distinct chatbot interfaces: a voice-only chatbot with anthropomorphic features and a text-only chatbot.<br><br>This design allows for a direct assessment of the impact of voice and anthropomorphic features on user experience.<br><br>Allocation Ratio:<br><br>Participants were randomly assigned to either the voice-only or text-only chatbot group in a 5:4 ratio. This means that for every 5 participants assigned to the voice-only group, 4 participants were assigned to the text-only group. This allocation ratio ensures a balanced distribution of participants between the two groups.		
**3b) CONSORT: Important changes to methods after trial commencement (such as eligibility criteria), with reasons**		
As there were no changes to the system, this item is not relevant for this study.		
**3b-i) Bug fixes, Downtimes, Content Changes**		
Not relevant for this study.		
**4a) CONSORT: Eligibility criteria for participants**		
Yes, the entire section is present in the paper. This will be demonstrated in the subitems.		
**4a-i) Computer / Internet literacy**		
The methods section does not explicitly state whether computer or internet literacy was an eligibility criterion. However, it can be inferred that participants were required to have a basic level of computer and internet literacy, as the study involved interacting with chatbots through a computer or mobile device.		
**4a-ii) Open vs. closed, web-based vs. face-to-face assessments:**		
The participants were notified that it was an on-site-face-to-face experiment in the recruitment ad.<br><br>"Our recruitment announcement, disseminated through various social media channels<br>associated with Sahlgrenska University's official account, specified that participants should be 18 years or older, free from any current mental health disorders, and willing to physically attend the testing facility in Gothenburg, Sweden."		
**4a-iii) Information giving during recruitment**		
"Our recruitment announcement, disseminated through various social media channels<br>associated with Sahlgrenska University's official account, specified that participants should be 18 years or older, free from any current mental health disorders, and willing to physically attend the testing facility in Gothenburg, Sweden."<br><br>"Each participant was required to provide informed consent before undergoing the<br>Generalized Anxiety Disorder Scale (GAD-7) assessment for anxiety symptoms. Those scoring 14 or higher on the GAD-7 were excluded from the study (Figure 2). Eligible participants were then randomly assigned to one of two groups: (1) engaging in text-based conversations with the text-onlyBETSY or (2) participating in voice-based interactions with the voice-only BETSY (Figure 2)"		
**4b) CONSORT: Settings and locations where the data were collected**		
Yes, the checklist item 4b is fulfilled and will be detailed in the following sections.		
**4b-i) Report if outcomes were (self-)assessed through online questionnaires**		
The methods section explicitly states that the outcomes were self-assessed through face-to-face data collection.<br><br>e.g. "participants were instructed to complete a questionnaire. This questionnaire covered their prior experiences with mental health chatbots as well as their demographic information, including sex, occupation, and marital status. Additionally, participants rated their overall well-being on a visual analog scale (VASW) ranging from 1 (not good at all) to 10 (feeling excellent) before starting their session with BETSY. "		
**4b-ii) Report how institutional affiliations are displayed**		

The methods section does not explicitly state how institutional affiliations were displayed to potential participants. The performing institute is relatively unknown and there is no prestige connected to the research group, however, if needed this will be added to the manuscript-

5) CONSORT: Describe the interventions for each group with sufficient details to allow replication, including how and when they were actually administered

5-i) Mention names, credential, affiliations of the developers, sponsors, and owners

The methods section mention the names, credentials, or affiliations of the developers, sponsors, or owners of the BETSY chatbot. It also names the systems used in order to make it more feasible to replicate.

"Two versions of the chatbot (Figure 1) were created: one enabling voice interaction with a
facial expression and an avatar component, and another relying solely on text-based
communication with an avatar image. The voice-only BETSY chatbot was implemented using Dialog Flow (Google) for conversation logic and connected to the UNEEQ platform for the human-avatar interface. Data infrastructure was hosted by Deloitte Digital and
VästraGötalandsregionen/VGR-IT. In contrast, the text-only BETSY chatbot was developed on the Itsalive.io platform and deployed to a research and development account on Facebook. Importantly, no personal metadata was collected during on-site testing via digital platforms."

5-ii) Describe the history/development process

The methods section provides a brief overview of the history and development process of the BETSY chatbot.

"This project adopted a participatory design approach to ensure broad involvement of
healthcare professionals, patients, and the public. A multidisciplinary team consisting of two
psychiatrists, two psychiatric nurses, four clinical psychologists, one patient, and one engineer was assembled to comprehensively address ethical, medical, and legal considerations for a potential chatbot. Team members were selected for their expertise in digitalization and psychiatry. Before the initial workshop, where the algorithm's preliminary outline was presented, the engineer created a survey. This survey drew partly from Radziwill and Benton's Quality Attribute listing [5], which synthesized findings from various chatbot usability projects."

Two versions of the chatbot (Figure 1) were created: one enabling voice interaction with a
facial expression and an avatar component, and another relying solely on text-based
communication with an avatar image. The voice-only BETSY chatbot was implemented using Dialog Flow (Google) for conversation logic and connected to the UNEEQ platform for the human-avatar interface. Data infrastructure was hosted by Deloitte Digital and
VästraGötalandsregionen/VGR-IT. In contrast, the text-only BETSY chatbot was developed on the Itsalive.io platform and deployed to a research and development account on Facebook. Importantly, no personal metadata was collected during on-site testing via digital platforms. Both versions of BETSY encompassed 24 topics (detailed in Appendix 1) related to mental health, including anxiety, depression, stress, sleep, addiction, eating disorders, anger, hopelessness, helplessness, loneliness, sadness, suicidal ideation, and suicidality, among others. These chatbots were designed in the Swedish language. An assessment was conducted to evaluate the alignment of the text-only and voice-only algorithms. Specifically, testers posed identical questions to both systems within various domains, with only one instance revealing a discrepancy when the voice-only chatbot could not provide an appropriate response while the text-based bot could, indicating the need for further refinement"

5-iii) Revisions and updating

This item is not relevant to this study as the system was a prototype with a single use approach. Only one singular version of the system was used. The chatbot did not undergo any bug fixes and updates during the course of the study.

5-iv) Quality assurance methods

The chatbot underwent rigorous testing to ensure that it functioned as intended and that the information it provided was accurate and up-to-date, this was present in the paper.

"An assessment was conducted to evaluate the alignment of the text-only and voice-only algorithms. Specifically, testers posed identical questions to both systems within various domains, with only one instance revealing a discrepancy when the voice-only chatbot could not provide an appropriate response while the text-based bot could, indicating the need for further refinement."

The study could however benefit from more detailed quality endurance descriptions as the study states that it was quality tested by experts.

" A multidisciplinary team consisting of two psychiatrists, two psychiatric nurses, four clinical psychologists, one patient, and one engineer was assembled to comprehensively address ethical, medical, and legal considerations for a potential chatbot. Team members were selected for their expertise in digitalization and psychiatry."

**5-v) Ensure replicability by publishing the source code, and/or providing screenshots/screen-capture video, and/or providing flowcharts of the algorithms used**		
The BETSY chatbot conversational items are available in the appendix, the URL to the chatbot appearance and functions will also be available in a repository. The full algorithm is owned by the university as proprietary knowledge, and this not available as source code.<br><br>"Both versions of BETSY encompassed 24 topics (detailed in Appendix 1) related to mental health, including anxiety, depression, stress, sleep, addiction, eating disorders, anger, hopelessness, helplessness, loneliness, sadness, suicidal ideation, and suicidality, among others."		
**5-vi) Digital preservation**		
This is not fully applicable to this study.		
**5-vii) Access**		
Participants accessed the BETSY chatbot through a web browser on an on-site computer provided by the researchers. They were not required to pay for access to the chatbot, and they did not need to be a member of any specific group. This is stated in the article:<br><br>" deployed to a research and development account on Facebook.<br>Importantly, no personal metadata was collected during on-site testing via digital platforms."		
**5-viii) Mode of delivery, features/functionalities/components of the intervention and comparator, and the theoretical framework**		
Mode of delivery was stated in the paper. The BETSY chatbot was a web-based application that can be accessed through web browser. As the system was only used once, on site, by the participants, the method only specifies mode of delivery and tracking during the on-site session.		
**5-ix) Describe use parameters**		
Several parameters were described e.g. the max lenght of the conversations as well as the procedures for EEG and BP measures.<br><br>"Before starting the chat with BETSY, participants were outfitted with a mobile dry-sensor EEG device to record their brain wave activity. Additionally, their blood pressure and pulse were recorded on the left arm after a 5-minute seated rest. Systolic and diastolic blood pressure were measured using a digital sphygmomanometer, and pulse was monitored with a pulse oximeter."<br><br>"Each participant had a maximum of 30 minutes to engage with the chatbot version they were assigned to"		
**5-x) Clarify the level of human involvement**		
There was no human involvement in the delivery of the BETSY chatbot intervention. Participants interacted with the chatbot independently.<br><br>"Each participant sat alone in the room, with the tester observing remotely via a non-recordable streaming camera. This camera served to facilitate real-time communication and allowed the tester to monitor the participant's reactions and anticipate any need for assistance. The participants were made aware of this procedure."		
**5-xi) Report any prompts/reminders used**		
As the system was a one-time use of a prototypes, no notifications or nudging was used during the 30 minutes they were conversing with the chatbot. They were however provided a helpful sheet that instructed them in which topics BETSY was developed in and how they could use these topics to move forward in the conversation.<br><br>"Participants were given instructions by the tester along with an accompanying sheet (see Appendix 2), which provided potential chat scenarios and specified the topics within BETSY's scope."		
**5-xii) Describe any co-interventions (incl. training/support)**		
Participants did not receive any co-interventions in addition to the BETSY chatbot intervention.		
**6a) CONSORT: Completely defined pre-specified primary and secondary outcome measures, including how and when they were assessed**		

The questionnaires were not used online, only in person and they were validated for both online and in person use. The main outcome: System Usability Scale is well versed and validated to the evaluation of usability in systems.

Other Questionnaires were validated, as they are widely used and have been shown to be reliable and valid in previous studies.

"While a universally accepted benchmark for conducting usability tests on chatbots remains elusive, numerous studies have gravitated toward the adoption of the System Usability Scale (SUS-10) [24- 29] and the Speech User Interface Service Quality (SUISQ) scale. SUS-10 captures the overall usability of a system independently of the platform or interface. The score ranges from 0 to 100, indicating higher usability with increasing score [26]. A score of 68 is considered as a passing grade, while a score below 50 is considered as indicating tha the system has less optimal usability. For a system to be considered as exceptionally good in terms of its design and usability, a score of 85 on average should be applied [29-31]."

6a-i) Online questionnaires: describe if they were validated for online use and apply CHERRIES items to describe how the questionnaires were designed/deployed

Not applicable for this study as the questionnaires were analogue and on-site.

6a-ii) Describe whether and how "use" (including intensity of use/dosage) was defined/measured/monitored

User metrics are reported in minutes per user. As this intervention focuses on usability and is a one-time use of a prototype, all participants used the system for a maximum and a minimum of 30 minutes.

"Each participant had a maximum of 30 minutes to engage with the chatbot version they were assigned to"

6a-iii) Describe whether, how, and when qualitative feedback from participants was obtained

Participants were given the opportunity to provide qualitative feedback through open-ended questions in the online questionnaires. This information is not essential for the study, as the primary and secondary outcomes are not directly related to qualitative feedback. These results will be published elsewhere.

"Furthermore, participants were provided with an open-ended questionnaire to gather their suggestions and insights regarding their session experience. It should be noted that qualitative data from this survey will be reported separately."

6b) CONSORT: Any changes to trial outcomes after the trial commenced, with reasons

Yes, the checklist item 4b is fulfilled and will be detailed in the following sections.

7a) CONSORT: How sample size was determined

7a-i) Describe whether and how expected attrition was taken into account when calculating the sample size

Sample size was based on a power calculation. The details of the power calculation are not present in the manuscript and should be added post-review.

7b) CONSORT: When applicable, explanation of any interim analyses and stopping guidelines

The questionnaires were not used online, only in person and they were validated for both online and in person use. The main outcome: System Usability Scale is well versed and validated to the evaluation of usability in systems.

Other Questionnaires were validated, as they are widely used and have been shown to be reliable and valid in previous studies.

"While a universally accepted benchmark for conducting usability tests on chatbots remains elusive, numerous studies have gravitated toward the adoption of the System Usability Scale (SUS-10) [24- 29] and the Speech User Interface Service Quality (SUISQ) scale. SUS-10 captures the overall usability of a system independently of the platform or interface. The score ranges from 0 to 100, indicating higher usability with increasing score [26]. A score of 68 is considered as a passing grade, while a score below 50 is considered as indicating tha the system has less optimal usability. For a system to be considered as exceptionally good in terms of its design and usability, a score of 85 on average should be applied [29-31]."

8a) CONSORT: Method used to generate the random allocation sequence

The methods section states that participants were randomized to either the voice-only or text-only chatbot group using a computer-generated random number generator. This is a standard and well-accepted method for generating random allocation sequences.<br><br>"The randomization process was conducted with strict double-blind procedures overseen by an independent researcher not affiliated with this project and facilitated by an automated randomization system, ensuring the impartiality of the allocation"<br><br>However, this is not a good description, as the system was not double-blind. The participants were fully aware of the system they were using, thus we need to change this statement post-review.		
**8b) CONSORT: Type of randomisation; details of any restriction (such as blocking and block size)**		
Simple randomization was used.		
**9) CONSORT: Mechanism used to implement the random allocation sequence (such as sequentially numbered containers), describing any steps taken to conceal the sequence until interventions were assigned**		
Allocation concealment was achieved through the use of a secure randomization system provided by a colleague not affiliated with the project.<br><br>"The randomization process was conducted with strict double-blind procedures overseen by an independent researcher not affiliated with this project and facilitated by an automated randomization system, ensuring the impartiality of the allocation"		
**10) CONSORT: Who generated the random allocation sequence, who enrolled participants, and who assigned participants to interventions**		
The research group did the enrolling, testing and announcing, this is stated in the manuscript. The randomisation is described in previous sections and was performed by a colleague not affiliated with the project.<br><br>This could be more clearly stated in the manuscript post-review. This is stated in the ICMJE declarations of the paper.		
**11a) CONSORT: Blinding - If done, who was blinded after assignment to interventions (for example, participants, care providers, those assessing outcomes) and how**		
**11a-i) Specify who was blinded, and who wasn't**		
It is not possible to blind participants in a trial with specific interfaces. However, assessors were blinded to the group assignment.		
**11a-ii) Discuss e.g., whether participants knew which intervention was the "intervention of interest" and which one was the "comparator"**		
The interventions were two different types of chatbots, and it is not possible to create a placebo or sham intervention for a chatbot.		
**11b) CONSORT: If relevant, description of the similarity of interventions**		
No. Not relevant for this trial.		
**12a) CONSORT: Statistical methods used to compare groups for primary and secondary outcomes**		
The methods section states that the primary and secondary outcomes were compared using linear regression analysis and t-tests. These are appropriate statistical methods for comparing two groups.<br><br>"All data was entered and processed in IBM SPSS version 28.0.1.1. For group differences, means analysis was employed using Pearson chi-square asymptotic significance (2-sided) set at .05 as significance level. For continuous outcome variables such as SUS-10, SUSIQ-MR, brain wave activity, positivity, and GAD-7, linear regression analyses were employed. The data was tested for kurtosis and skewness. Based on the results, t-test was performed. All results were analyzed according to group"		
**12a-i) Imputation techniques to deal with attrition / missing values**		
No imputation was applied. Missing values were presented as missing.		
**12b) CONSORT: Methods for additional analyses, such as subgroup analyses and adjusted analyses**		
Not applicable to this paper.		
RESULTS		
**13a) CONSORT: For each group, the numbers of participants who were randomly assigned, received intended treatment, and were analysed for the primary outcome**		
Not relevant for this study as it was healthy participants in a one-time test.		
**13b) CONSORT: For each group, losses and exclusions after randomisation, together with reasons**		

This is provided in a figure and in text.<br><br>"Of the 50 individuals who initially volunteered, five participants (2 men and 3 women) opted out before providing their consent (Figure 2). Subsequently, 45 individuals attended the screening at the test facility. Each participant was required to provide informed consent before undergoing the Generalized Anxiety Disorder Scale (GAD-7) assessment for anxiety symptoms. Those scoring 14 or higher on the GAD-7 were excluded from the study (Figure 2). Eligible participants were then randomly assigned to one of two groups: (1) engaging in text-based conversations with the text-only BETSY or (2) participating in voice-based interactions with the voice-only BETSY (Figure 2)"		
**13b-i) Attrition diagram**		
Not relevant for this study.		
**14a) CONSORT: Dates defining the periods of recruitment and follow-up**		
Recruitment was defined, follow-up not relevant as this was a one-time test.		
**14a-i) Indicate if critical “secular events” fell into the study period**		
Not relevant to this study.		
**14b) CONSORT: Why the trial ended or was stopped (early)**		
Not relevant to this study.		
**15) CONSORT: A table showing baseline demographic and clinical characteristics for each group**		
This is provided in Table 1 of the paper.		
**15-i) Report demographics associated with digital divide issues**		
Age, gender, education, socioeconomic status and general positivity and experience of use of technology was provided in Table 1 of the study.		
**16a) CONSORT: For each group, number of participants (denominator) included in each analysis and whether the analysis was by original assigned groups**		
**16-i) Report multiple “denominators” and provide definitions**		
In this study the n of x was noted for each of the group, and in connection to their use of the chatbot. As the study was one-time test, all participants used the software only once for the same length of time.		
**16-ii) Primary analysis should be intent-to-treat**		
Not relevant for this study.		
**17a) CONSORT: For each primary and secondary outcome, results for each group, and the estimated effect size and its precision (such as 95% confidence interval)**		
The study reports results for primary outcomes, such as self-reported emotional states, system usability (SUS-10), and biometric measures.<br><br>Additional information on process outcomes, such as metrics of use and intensity of use, is recommended for a more comprehensive presentation.		
**17a-i) Presentation of process outcomes such as metrics of use and intensity of use**		
This item is not fully relevant as dose-time differences do not exist, and the outcome is not treatment but exploration of interface among healthy volunteers.		
**17b) CONSORT: For binary outcomes, presentation of both absolute and relative effect sizes is recommended**		
This is not applicable in this study-		
**18) CONSORT: Results of any other analyses performed, including subgroup analyses and adjusted analyses, distinguishing pre-specified from exploratory**		
The study mentions the comparison of users in the analysis. It's highlighted that this is a self-selected sample, recognizing the potential bias introduced by this approach.		
**18-i) Subgroup analysis of comparing only users**		
As this was not a comparison of treatment and waiting list, all users were using the system in order to provide metrics for usability. Thus item 18 is not completely relevant for this trial.		
**19) CONSORT: All important harms or unintended effects in each group**		
Many of these aspects are theoretically discussed in the discussion. As this was not a trial of treatment, but exploration of usability in healthy participants, potential harm was not discussed in depth.		
**19-i) Include privacy breaches, technical problems**		

This was a one-time test, performed on site. No data or devices were used that belonged to the participants. There was no breach or meta-data collected.<br><br>"Importantly, no personal metadata was collected during on-site testing via digital platforms."		
**19-ii) Include qualitative feedback from participants or observations from staff/researchers**		
This was collected, as mentioned in previous sections, but will be published elsewhere.<br><br>"Furthermore, participants were provided with an open-ended questionnaire to gather their<br>suggestions and insights regarding their session experience. It should be noted that qualitative data from this survey will be reported separately."		
DISCUSSION		
**20) CONSORT: Trial limitations, addressing sources of potential bias, imprecision, multiplicity of analyses**		
**20-i) Typical limitations in ehealth trials**		
This is present in the paper.<br><br>"This study consisted of healthy volunteers. It is good to keep in mind that mental health issues can affect some parts of cognitive performance [48] and, thus, usability may not be equally perceived by a person in a state of emotional distress and a healthy volunteer. Further investigation and collaboration are needed in future studies to capture usability aspects of individuals who are in an active state of distres		
**21) CONSORT: Generalisability (external validity, applicability) of the trial findings**		
**21-i) Generalizability to other populations**		
**21-ii) Discuss if there were elements in the RCT that would be different in a routine application setting**		
**22) CONSORT: Interpretation consistent with results, balancing benefits and harms, and considering other relevant evidence**		
**22-i) Restate study questions and summarize the answers suggested by the data, starting with primary outcomes and process outcomes (use)**		
Yes, this is fully covered in the paper.<br><br>e.g. "While the text-only system scored higher on usability, both versions of the chatbot scored average or above average with respect to overall usability [31]. The mean text-only chatbot SUS-10 score of 75.34 falls between the threshold good (a score of 70) and excellent (a score of 80 and above) [29-31]. However, the score for the voice-only chatbot (64.8) indicates that the system is perceived to be usable, but has room for improvement. Usability can be affected by many factors such as user interface design, content layout, and overall user experience [42,43]. The voice-only chatbot score indicates that there may be areas for improvement in terms of all of aforementioned aspects. It should also be noted that the SUS-10 scale does not measure a specific feature or aspect of system design, but instead provides an overall assessment of user experience [31]. Using more elaborate scales that cover more dimensions across the system is more suitable for more in depth analysis of the usability of chatbots. It can also be noted that the range of scores was much higher for the text-only interface (lowest score for text-only group was 57 and the equivalent for the voice only chatbot was 40), which indicates much poorer usability"		
**22-ii) Highlight unanswered new questions, suggest future research**		
Yes. It highlights questions and propositions for future research.		
Other information		
**23) CONSORT: Registration number and name of trial registry**		
Not noted in manuscript. Will be added post-review.		
**24) CONSORT: Where the full trial protocol can be accessed, if available**		
https://www.youtube.com/watch?v=jfnS5zgNCRY&t=5s		
**25) CONSORT: Sources of funding and other support (such as supply of drugs), role of funders**		
Yes, it is noted in the paper.		
**X26-i) Comment on ethics committee approval**		

Ethical considerations and ethical approval is stated in this paper.<br><br>"All ethical decisions were guided by the Declaration of Helsinki and its subsequent amendments. The study protocol was reviewed and approved by the central ethical review board in Sweden (DRN 2021-02771). All precautions were taken in order to avoid any possible contagion from COVID-19. Due to the nature of the prototype, no patients were used in this initial examination of the chatbot in order to ensure that vulnerable participants would not be negatively affected by errors and flaws that might be present in a prototype-stage system."		
**x26-ii) Outline informed consent procedures**		
Informed consent was provided to the participants.<br><br>"Of the 50 individuals who initially volunteered, five participants (2 men and 3 women) opted out before providing their consent (Figure 2). Subsequently, 45 individuals attended the screening at the test facility. Each participant was required to provide informed consent before undergoing the Generalized Anxiety Disorder Scale (GAD-7) assessment for anxiety symptoms. Those scoring 14 or higher on the GAD-7 were excluded from the study (Figure 2)."		
**X26-iii) Safety and security procedures**		
Safety and security are clearly described in this paper, especially the precautions taken to avoid contaminating the participants with viral infections such as SARS-COV-2 and protect their metadata. The paper also mentions the rational behind using health participant to avoid vulnerable individuals being exposed to un-tested technology.<br><br>"Participants were greeted by a tester wearing protective gear, including a surgical R-II mask, gloves, a face visor, and hospital scrubs. Protective gear was also offered to participants upon their arrival. Participants were then placed in a sanitized room equipped with a screen, which underwent thorough sanitization with medical-grade disinfectants and a sterilizing UV lamp before and after each participant's session"<br><br>"A survey was distributed to the public via the secure research platform Psytoolkit.org, ensuring participants' anonymity by omitting the collection of metadata such as IP addresses and locations"<br><br>"In this initial phase of system exploration, our focus was on evaluating the system's capabilities using volunteers who did not exhibit severe anxiety. As the system is still in its prototype stage, we exercised caution to avoid any potential exacerbation of symptoms in individuals with severe anxiety."		
**X27-i) State the relation of the study team towards the system being evaluated**		
